# Supplementary material for: Multi-Omic Analysis of CIC’s Functional Networks Reveals Novel Interaction Partners and a Potential Role in Mitotic Fidelity
Source: Cancers (Basel). 2023 May 17;15(10):2805. doi: 10.3390/cancers15102805 (PMC10216487; doi:10.3390/cancers15102805)
Supplement: Supplementary file 1 [file cancers-15-02805-s001.zip › Supplementary_materials_merged.pdf]

Supplementary Methods

Single-cell RNA-seq (scRNA-seq) library construction and sequencing

Cells were harvested using 0.05% Trypsin-EDTA, washed twice with 1x PBS + 0.04% BSA, and passed through a 35 µm strainer to generate a single-cell suspension. Single cell 3' RNA-seq libraries were generated using the Chromium Single Cell 3′ Library & Gel Bead Kit v1 (10X Genomics, Pleasanton, CA, USA) following the manufacturer's protocol. Briefly, the volume of cell suspension was determined based on a targeted cell recovery of 1,000 cells and then mixed with the Single Cell Master Mix prior to loading onto the Single Cell 3' Chip. Following gel bead in emulsion (GEM) generation in the Chromium Controller (10X Genomics), the GEM-RT reaction was performed, GEMs were broken, and cDNA clean up was performed using Dynabeads™ MyOne Silane beads (Thermo Fisher Scientific, Waltham, MA, USA) followed by a cleanup with SPRIselect beads (Beckman Coulter, Indianapolis, IN, USA). cDNA amplification was performed using a total of 14 amplification cycles, followed by reaction clean up with SPRIselect beads. Total cDNA quantification was performed using the Agilent High Sensitivity DNA Kit (Santa Clara, CA, USA). cDNA shearing on the Covaris E220 Focused Ultrasonicator (Woburn, MA, USA), library construction (10 cycles for sample index PCR), and SPRIselect cleanups were performed to generate the final indexed libraries. Library quality was assessed using the Agilent High Sensitivity DNA Kit and quantified using the Qubit dsDNA HS Assay Kit (Thermo Fisher Scientific). Libraries were pooled (8 libraries/pool) and sequenced on one flowcell on the Illumina NextSeq500 platform (San Diego, CA, USA) using paired-end sequencing with dual indexing following Illumina protocols and 10X sequencing run parameters (98bp read1, 14bp i7 index, 8bp i5 index, and 98bp read2).

Cell lysis preparations

Cell pellets were resuspended in 4x packed cell volume of RIPA lysis buffer freshly supplemented with 1% sodium orthovanadate (100mM), 1% PMSF (200mM), 2% (v/v) protease inhibitor (sc-24948; Santa Cruz Biotechnology, Dallas, TX, USA), and PhosSTOP (Roche, following the manufacturer's recommendations). Cell pellets were sonicated briefly and mixed for 1 h at 4°C on a rotator. Cellular debris was removed using centrifugation at 13,000 rpm for 10 min at 4°C. Total protein was quantified using the Pierce™ BCA Protein Assay Kit (Thermo Fisher Scientific).

Western blots

Samples were subjected to gel electrophoresis on NuPage® 3-8% Tris Acetate or 10% Bis-Tris pre-cast mini-gels with 1x NuPage® MOPS buffer (Thermo Fisher Scientific). Separated proteins were transferred onto a methanol-activated PVDF membrane (162-0177; Bio-Rad Laboratories, Hercules, CA, USA) in 1x NuPage® Transfer buffer (Thermo Fisher Scientific) + 20% (v/v) methanol. Membranes were blocked with either 2% or 5% (w/v) skim milk in PBST or ReliaBLOT BLOCK (WB 120; Bethyl Laboratories, Montgomery, TX, USA) in TBST for 1 h at room temperature prior to incubation with primary antibodies (Supplementary Table S10) at 4°C overnight. For protein detection, membranes were incubated with HRP-IgG goat α-mouse or α-rabbit (1:5000; Santa Cruz Biotechnology), or ReliaBLOT α-Rabbit HRP (1:5000; Bethyl Laboratories) for 1 h at room temperature followed by three PBST washes before application of ECL substrate (Bio-Rad Laboratories) or SuperSignal West Femto substrate (Thermo Fisher Scientific). Images were captured using a ChemiDoc™ MP Imager and processed with Protein Image Lab 5.1 (Bio-Rad Laboratories).

Nuclear fractionation and immunoprecipitation

Cells were grown for 48 h and harvested at ~80% confluency. Fresh cell pellets were gently resuspended in 5x packed cell volume of cytoplasmic lysis buffer (10mM Tris-HCl pH7.2, 10mM NaCl,

2mM $MgCl_2$, 1mM EDTA, 0.05% NP-40, with 1X cOmplete protease inhibitor [Roche] and 1X PhosSTOP [Roche]). Lysates were incubated on ice for 10 min before centrifugation at 300g for 5 min to collect nuclear pellets. After 3 to 4 washes with wash buffer (10mM Tris-HCl pH7.2, 10mM NaCl, 2mM $MgCl_2$, 1mM EDTA with 1X cOmplete protease inhibitor and 1X PhosSTOP), nuclear pellets were resuspended in 3x packed cell volume of nuclear lysis buffer (250mM NaCl, 20mM sodium phosphate pH7.0, 30mM sodium pyrophosphate, 5mM EDTA, 10mM sodium fluoride, 10% glycerol, 1% NP-40, 1mM DTT, with 1X cOmplete protease inhibitor and 1X PhosSTOP) and then homogenized using a 21-gauge needle. Cellular debris was removed by centrifugation at 13,000 rpm for 30 min at 4°C and then nuclear protein was quantified using the Pierce™ BCA Protein Assay Kit (Thermo Fisher Scientific). Before proceeding with immunoprecipitation, nuclear lysates were treated with 0.1X Benzonase (MilliporeSigma, Burlington, MA, USA) for 30 min at 4°C. Antibody-bound magnetic beads were prepared by incubating anti-CIC or normal rabbit-IgG antibodies (Supplementary Table S10) with Protein G Dynabeads^{TM} (Thermo Fisher Scientific) in PBST (0.1% v/v) at 4°C for 30 min, and then rinsed three times with wash buffer (1X PBS, 1mM EDTA, 0.5% NP40, with 1X cOmplete protease inhibitor and 1X PhosSTOP). Nuclear lysates were incubated with 1.5 mg of the prepared anti-CIC or rabbit IgG (control) Protein-G beads at 4ºC overnight. Protein and protein complexes were released by boiling the magnetic beads in the elution buffer (2X Nupage LDS buffer [Thermo Fisher Scientific], 200mM DTT) at 98ºC for 10 min.

Reciprocal immunoprecipitation

Whole-cell extracts were prepared by lysing fresh cell pellets with 2x packed cell volume of lysis buffer (25mM Tris-HCl pH7.4, 150mM NaCl, 1% NP-40, 5% glycerol, with 1X cOmplete protease inhibitor [Roche] and 1X PhosSTOP [Roche]), followed by homogenization using 10 passes through a 21-gauge needle. After a 30 min incubation on ice, lysis preparations were cleared of cellular debris by centrifugation at 13,000 rpm for 30 min at 4°C and resuspended in 200 ul of lysis buffer. Whole-cell lysates were incubated with anti-ARID1A, anti-ARID2, anti-SMARCA2, anti-SMARCC2, or mouse/rabbit-IgG antibodies

(Supplementary Table S10) at 4ºC overnight. Proteins and complexes were captured by incubating antibody-lysate mixtures with Protein G Dynabeads^{TM} for 1 h at 4°C. For elution, magnetic beads were boiled in elution buffer (2X Nupage LDS buffer, 200mM DTT) at 98ºC for 10 min.

HEK cell lines MS data analysis

Data from the 4000 QTrap were processed using Mascot Software (v2.5.1) [149]. MS2 spectra were searched against the Uniprot-Swissprot database (v2020March) [150,151] using the Homo sapiens taxonomy filter (20,366 total entries). Mascot parameters were specified as: trypsin enzyme, 1 missed cleavage allowed, peptide mass tolerance of 0.8 Da, and a fragment mass tolerance of 0.8 Da. Oxidation of methionine and deamidation at NQ were set as variable modifications. Carbamidomethylation of cysteine was set as a fixed modification. An ion score cutoff of 34 and required bold red criteria were used to filter the protein hits.

NHA cell lines LC-MS/MS analysis

<u>Protein elution, clean-up with SP3, and protease digestion</u>

Proteins eluted from IP preparations from NHA lines in SDS loading buffer were purified using the SP3 method, as described previously [152,153]. A 1:1 combination of two different types of carboxylate-functionalized beads, both with a hydrophilic surface (Sera-Mag Speed Beads, 45152105050350 and 65152105050350; GE Life Sciences, Chicago, IL, USA) was added to the lysate and ethanol was added to achieve a final concentration of 50% by volume. Tubes were mixed on a ThermoMixer unit (Eppendorf, Hamburg, Germany) at 1,000 rpm for 10 min at room temperature, then placed in a magnetic rack and incubated for 2 min. The supernatant was discarded, and the beads were rinsed 3x with 180 μL of 90% ethanol by removing the tubes from the magnetic rack and gently re-suspending the beads by pipette mixing. For elution, the tubes were removed from the magnetic rack and beads were resuspended in 100 μL of 50 mM HEPES (pH 8) containing an appropriate amount of trypsin/rLysC mix (1:25 enzyme

to protein concentration; V5071; Promega, Madison, WI, USA) and incubated for 14 h at 37°C in a ThermoMixer with mixing at 1000 rpm. After incubation, the tubes were sonicated briefly (30 s) in a bath sonicator, placed on a magnetic rack, and the supernatant was recovered for further processing.

Synthetic peptide mix preparation

The set of standard peptides was taken as a subset from the collection analys ed in the ProteomeTools initiative [154]. Peptides were selected for a panel of 51 proteins, resulting in a set of 254 total candidates that were synthesis ed in a 'SpikeMix' format (Supplementary Table S6; JPT Peptide Technologies, Berlin, Germany). Upon delivery, dried peptides were reconstituted in 100 μL of DMSO, vortexed briefly (~15 s), and sonicated in a water bath for 5 min. Reconstituted peptides were measured in a dilution series to determine the concentration that represented the limit of detection for the majority of the pool. Reconstituted peptides were spiked into interactome samples at a concentration 10% above the determined limit of detection. In this way, the synthetic spikes would not negatively impact the resulting quantification of detected peptides due to a large difference in dynamic range in comparison to the IP samples.

Tandem mass tag (TMT) labelling of peptides

TMT 6-plex labelling kits were obtained from Pierce (Thermo Fisher Scientific). Each TMT label (5 mg per vial) was reconstituted in 500 μL of acetonitrile and refrozen. Labelling reactions were carried out through addition of 200 μg of TMT label in two volumetrically equal steps of 10 μL (100 μg per addition), 30 min apart. Control IP samples were labelled with TMT 126C, 127N, and 128C. Target IP samples were labelled with TMT 129N, 130C, and 131N. The synthetic peptide spikes were labelled using the TMT131C reagent (from the TMT 11-plex reagent set). Reactions were quenched by the addition of 10 μL of glycine (1M stock solution, Sigma-Aldrich). Labelled peptides were concentrated on a SpeedVac

centrifuge (Thermo Fisher Scientific) to remove excess acetonitrile, acidified to 1% (v/v) trifluoroacetic acid (TFA), and purified with C18 StageTips (Thermo Fisher Scientific).

Peptide clean-up procedures

Peptides were desalted and concentrated using StageTip treatment as described previously [155]. For StageTip clean-up, three discs of C18 Empore material (66883-U; Sigma-Aldrich) packed in 200µL pipette tips were rinsed twice with 100 µL of acetonitrile with 0.1% TFA. Cartridges were then rinsed twice with 100 µL of water with 0.1% TFA prior to sample loading. Loaded samples were rinsed twice with 0.1% formic acid (100 µL per rinse) and eluted with 100 µL of 80% acetonitrile containing 0.1% formic acid. All TopTip- or StageTip-processed samples were concentrated in a SpeedVac centrifuge (Thermo Fisher Scientific) and subsequently reconstituted in 1% formic acid with 1% DMSO in water.

MS analysis of peptide samples on the Orbitrap Fusion

Analysis of TMT-labelled peptide pools was carried out on an Orbitrap Fusion Tribrid MS platform (Thermo Fisher Scientific). Samples were introduced using an Easy-nLC 1000 system (Thermo Fisher Scientific). Columns used for trapping and separations were packed in-house. Trapping columns were packed in 100 µm internal diameter capillaries to a length of 25 mm with C18 beads (Reprosil-Pur, 3 µm particle size; Dr. Maisch HPLC GmbH, Ammerbuch, Germany). Trapping was carried out for a total volume of 10 µL at a pressure of 400 bar. After trapping, gradient elution of peptides was performed on a C18 column (Reprosil-Pur, 1.9µm particle size; Dr. Maisch HPLC GmbH) packed in-house to a length of 20 cm in 100 µm internal diameter capillaries with a laser-pulled electrospray tip and heated to 50°C using AgileSLEEVE column ovens (Analytical Sales & Services, Flanders, NJ, USA). Elution was performed with a gradient of mobile phase A (water and 0.1% formic acid) to 8% B (acetonitrile and 0.1% formic acid) over 5 min, to 30% B over 88 min, and to 40% B over 19 min, with final elution (80% B) using a further 8 min at a flow rate of 350 nL/min.

Data acquisition on the Orbitrap Fusion was carried out using a data-dependent method with multi-notch synchronous precursor selection MS3 scanning for TMT tags. Survey scans covering the mass range of 350 – 1500 were acquired at a resolution of 120,000 (at m/z 200), with quadrupole isolation enabled, an S-Lens RF Level of 60%, a maximum fill time of 50 ms, and an automatic gain control (AGC) target value of 5e5. For MS2 scan triggering, monoisotopic precursor selection was enabled, charge state filtering was limited to 2 – 4, an intensity threshold of 5e3 was employed, and dynamic exclusion of previously selected masses was enabled for 60 s with a tolerance of 20 ppm. MS2 scans were acquired in the ion trap in Rapid mode after CID fragmentation with a maximum fill time of 150 ms, quadrupole isolation, an isolation window of 1.6 m/z (0.2 m/z offset), collision energy of 30%, activation Q of 0.25, injection for all available parallelizable time turned OFF, and an AGC target value of 4e3. Fragment ions were selected for MS3 scans based on a precursor selection range of 400-1600 m/z, ion exclusion of 20 m/z low and 5 m/z high, and isobaric tag loss exclusion for TMT. The top 10 precursors were selected for MS3 scans that were acquired in the Orbitrap after HCD fragmentation (NCE 60%) with a maximum fill time of 150 ms, 50,000 resolution, 120-750 m/z scan range, ion injection for all parallelizable time turned OFF, and an AGC target value of 1e5. The total allowable cycle time was set to 4 s. MS1 and MS3 scans were acquired in profile mode, and MS2 in centroid format.

NHA cell lines MS data analysis

Data from the Orbitrap Fusion were processed using the Proteome Discoverer Software (v2.1.1.21). MS2 spectra were searched using Sequest HT against a combined UniProt human proteome database appended to a list of common contaminants (24,624 total sequences). Sequest HT parameters were specified as: trypsin enzyme, 2 missed cleavages allowed, minimum peptide length of 6, precursor mass tolerance of 20 ppm, and a fragment mass tolerance of 0.6. Oxidation of methionine and TMT 6-plex at lysine and peptide N-termini were set as variable modifications. Carbamidomethylation of cysteine was set as a fixed modification. Peptide spectral match error rates were determined using the target-decoy strategy coupled

to Percolator modelling of positive and false matches [156,157]. Data were filtered at the peptide spectral match-level to control for false discoveries using an adjusted *p*-value cut off of 0.01 as determined by Percolator. Contaminant and decoy proteins were removed from all data sets prior to downstream analysis.

Immunofluorescence assays

For cell lines, fixed cells were permeabilized using 0.2% TritonX-100/PBS for 5 min, blocked with 2.5% goat serum (G6767; Sigma-Aldrich) in PBS for 1 h on a rocker, and stained with appropriate primary antibodies (Supplementary Table S13) in 0.25% goat serum in PBS overnight at 4°C. Alexa Fluor® 488 and/or 568 (Thermo Fisher Scientific) were used as secondary antibodies (Supplementary Table S13), followed by mounting with DAPI (D3571; Thermo Fisher Scientific) and SlowFade™ Diamond Antifade Mountant (36967; Thermo Fisher Scientific).

For mouse tissue, embryonic (E13.5) mouse brain section slides were quickly pre-washed in 1X TBST (0.1% Tween) followed by a 15 min permeabilization in TBST (0.25% Tween) and an additional 5 min wash in TBST (0.1% Tween) with gentle shaking. Slides were blocked with 3% goat serum in 1X PBST (0.1% Tween), covered with parafilm, for 30 min in a humidified staining chamber. α-Phospho Histone H3 (Cell Signaling Technology, Danvers, MA, USA) and α-Rabbit Alexa Fluor® 568 (Thermo Fisher Scientific) antibodies were applied in blocking solution overnight at 4°C and for 1 h at room temperature, respectively, with three 5 min TBST (0.1% Tween) washes performed in between. Slides were then mounted for imaging and quantification. All steps were performed at room temperature unless indicated otherwise.

Proximity ligation assays (PLAs)

Cells were plated on an 8-well CC_2 chamber slide with cover (12-565-1; Fisher Scientific) in growth media. After 19 h, cells were fixed with 4% PFA. PLAs were performed using the Duolink® In Situ Red Starter Kit Mouse/Rabbit (DUO92101; Sigma-Aldrich), following the manufacturer's protocol. High-

resolution images were obtained with an Eclipse Ti inverted confocal microscope equipped with A1 si laser (Nikon, Tokyo, Japan) and Rolera EM-C^{2TM} (Teledyne QImaging, Surrey, BC, Canada) camera. Images used for quantifications were obtained with an Axio Observer inverted fluorescent microscope (Zeiss, Oberkochen, Germany), equipped with Apotome.2 and AxioCam MRm camera. Images were obtained at 40x or 63x (oil) magnification using the Zen software (2.3, blue edition; Zeiss). PLA dots were counted using Blob-Finder software (http://www.cb.uu.se/~amin/BlobFinder/).

Human chromosomal defect counts

To quantify cells for metaphase defects, 20-25 images per replicate were captured with 40x magnification. Normal, minor defective (a few alignment defects), and moderate-severe defective (severe alignments and multipolar alignments) phenotypes were observed. The proportion of cells with a defective phenotype (number of cells with defective phenotypes in metaphase / total number of cells in metaphase) was calculated for each image. Similarly, for cytokinesis defects, 50 images per replicate were captured with 63x (oil) magnification and the proportion of cells with a defective phenotype (number of cells with defective phenotypes in cytokinesis / total number of cells in cytokinesis) was calculated for each image. We did not observe a sufficient number of cells in cytokinesis in the HEK lines to quantify defects. All images were captured with a Zeiss Axio Observer with Apotome.2 fluorescence microscope.

Mouse chromosomal defect counts

E13.5 mouse brain [106] section slides were mounted on glass slides and stored at −80°C until analysis. Each slide contained four consecutive sections each from two mice, for a total of eight sections per slide. α-tubulin (Abcam, Cambridge, UK) and DAPI staining were used to quantify chromosomal defects and α-phospho-Histone H3 (Cell Signaling Technology) staining was used to visualis e mitotically active cells. For each brain section, images from 10 regions with cells in anaphase and telophase were captured at 63x (oil) magnification using an Axioplan microscope. The proportion of cells with defective

anaphase or telophase to the total number of cells at anaphase or telophase, respectively, was used for quantifications.

Microscopy imaging

Microscope information is included in relevant sections. Raw images were further processed in Photoshop (Adobe, Mountain View, CA, USA).

Enrichment analysis and gene function annotation

We used (v3.16.1) (Wu et al. 2021) to identify pathway and protein complexes significantly enriched (BH-adjusted p-value < 0.05) in *CIC* genetic interactors and protein interactors, as well as to annotate functions of gene affected by splicing events with GO biological pathways (unadjusted p-value < 0.05). Given many related GO terms with similar sets of genes associated with them, we calculated the degree of overlapping genes associated between GO terms using the Jaccard index, then summarised them into distinct groups by performing hierarchical clustering of the Jaccard index between pairs of GO terms. The number of distinct clusters was determined using the gap statistic, which calculated the optimal number of clusters (up to 30 clusters) by iteratively bootstrapping 1000 times using the cluster (v2.1.4).

Supplementary Figures

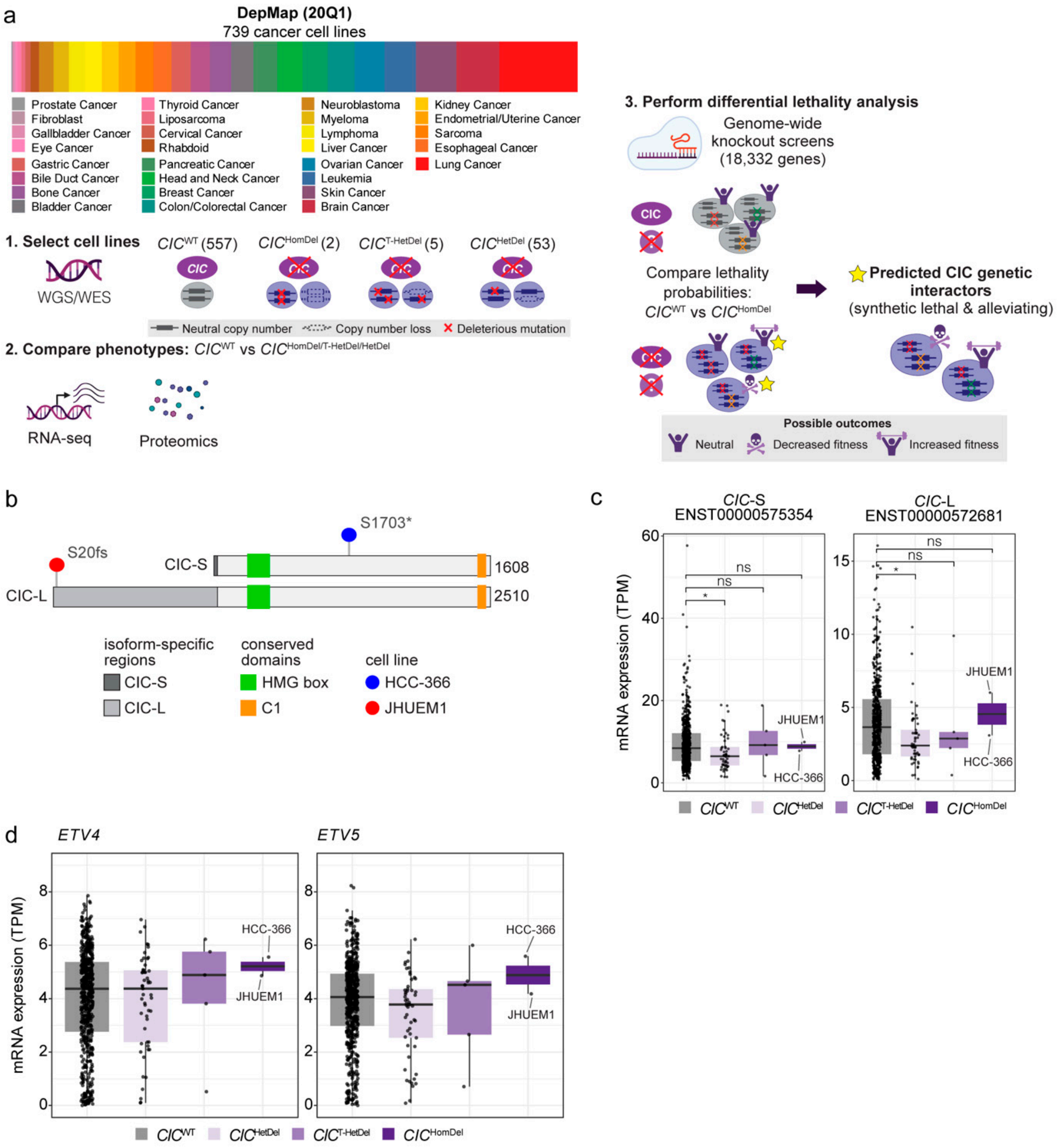

Supplementary Figure S1: Workflow of *CIC* in silico genetic interaction screen leveraging data generated by DepMap.

a. Workflow schematic. Cancer cell lines harbouring WT *CIC* alleles (CIC^{WT}) and mutant *CIC* alleles (CIC^{HomDel}, $CIC^{T\text{-}HetDel}$, and CIC^{HetDel}) were identified using mutation and copy number data from DepMap. mRNA and protein expression data were used to compare *CIC* mutant cell line phenotypes to CIC^{WT} phenotypes. The lethality probabilities across 18,333 genes were used to perform Mann-Whitney U-based differential lethality analyses to identify genes with significantly higher or lower lethality probabilities in the CIC^{HomDel} cell lines compared to CIC^{WT} cell lines, which indicate SL or alleviating genetic interactions with *CIC*, respectively. **b.** *CIC* gene model showing the homozygous deleterious alterations identified in both CIC^{HomDel} cell lines. **c-d.** *CIC*-S and *CIC*-L isoform transcripts and *ETV4* and *ETV5* total mRNA

abundance in *CIC*^{HomDel} (n = 2), *CIC*$^{\text{T-HetDel}}$ (n = 5), *CIC*^{HetDel} (n = 53), and *CIC*^{WT} (n =557) cancer cell lines. ANOVA followed by Tukey's HSD analysis. *p-value < 0.05 and ns > 0.05.

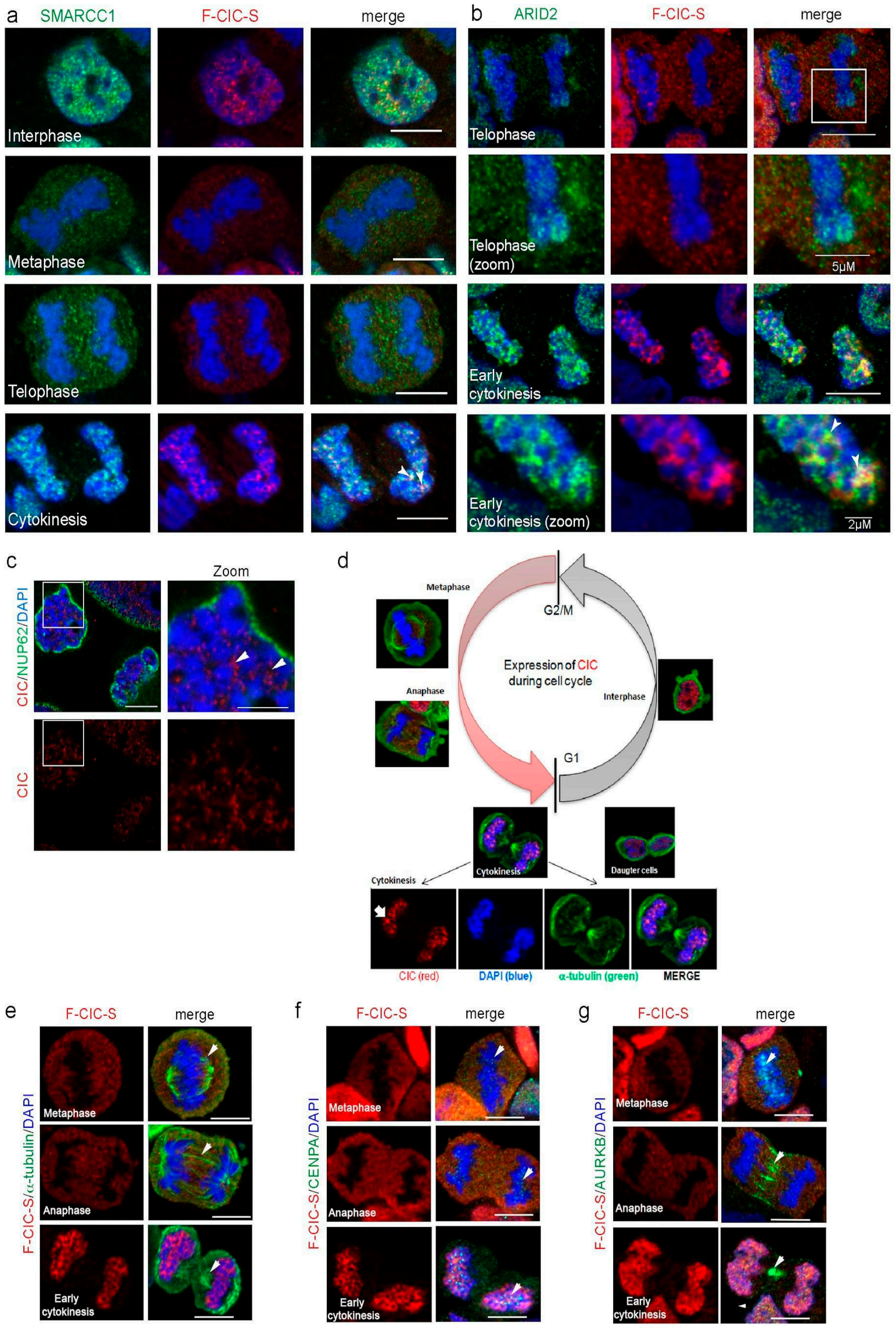

Supplementary Figure S2: Nuclear CIC colocalis___es with members of the SWI/SNF complex and is dynamically re-distributed during the cell cycle.

a-b. IF images show colocalis ation of F-CIC-S (FLAG, red) and SMARCC1 (a) or ARID2 (b, both green) in HEK-$CIC^{KO\text{-}D10}$ $^{+F\text{-}CIC\text{-}S}$ cells at indicated phases of the cell cycle. DNA was stained with DAPI (blue). Arrowheads indicate colocalis ation of CIC and the relevant interactor at early cytokinesis (yellow foci). Scale bars: 10 µm or as indicated for zoomed images (b). **c.** Expression of endogenous CIC (red) and the nuclear envelope protein NUP62 (green) in HOG-CIC^{WT} cells at cytokinesis. DNA was detected using DAPI staining (blue). Zoomed image of early cytokinesis (right) shows a punctate localization pattern for endogenous CIC throughout the decondensing nucleus (arrowheads). Scale bars: 10 µm and 5 µm (zoomed image). **d**. Expression of F-CIC-S (FLAG, red) and α-tubulin (green) in HEK-$CIC^{KO\text{-}D10}$ cells stably expressing F-CIC-S during different stages of the cell cycle. Foci of CIC protein can be observed adjacent to decondensing chromosomes at early cytokinesis (arrow, bottom panel). **e-g** IF images showing no colocalis ation of F-CIC-S (FLAG, red) with spindles (marked by α-tubulin, e), centrosomes/kinetochores (marked by CENPA, f), or midbody structures (marked by AURKB, g) in HEK-$CIC^{KO\text{-}D10\,+F\text{-}CIC\text{-}S}$ cells. Arrowheads indicate the structures of interest (markers in green), and DNA was stained with DAPI (blue). Representative images are shown for cells in metaphase, anaphase, and early cytokinesis. Scale bar: 10 µm.

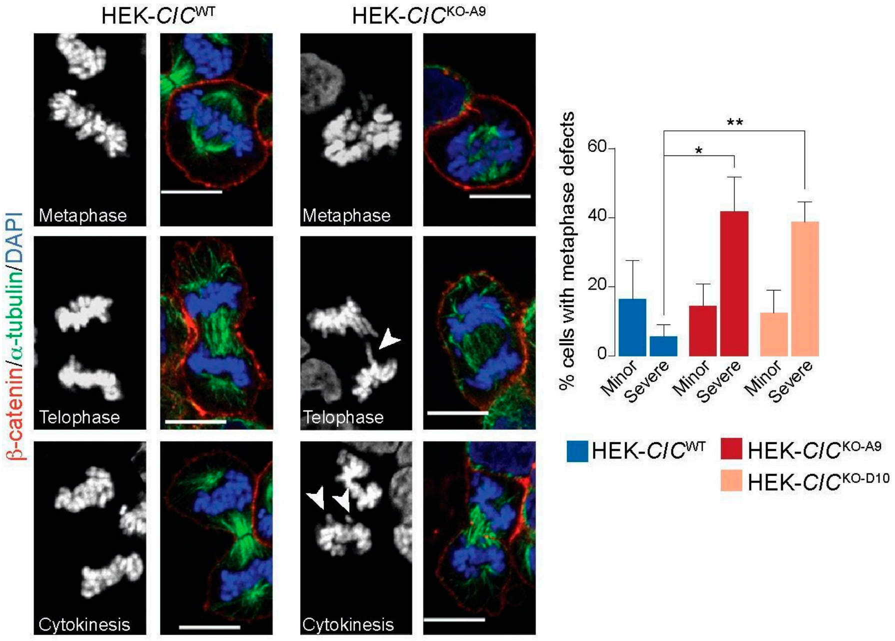

Supplementary Figure S3: Loss of CIC is associated with an increased frequency of mitotic defects.

Left: representative IF images from HEK-CIC^{WT} and HEK-$CIC^{KO\text{-}A9}$ cells at metaphase and telophase/cytokinesis. Microtubules (α-tubulin, green), DNA (DAPI, blue) and the cell membrane (ß-catenin, red) were detected. DAPI staining alone is shown on the left of each image. HEK-$CIC^{KO\text{-}A9}$ cells show defects in metaphase alignment and lagging chromosomes at telophase and cytokinesis (arrowheads). Scale bar: 10 µm. Right: bar graphs show proportions of cells with defects at metaphase. Bars represent the mean of three independent experiments and error bars indicate s.e.m. HEK-CIC^{WT}, n = 59; HEK-$CIC^{KO\text{-}A9}$, n = 61; HEK-$CIC^{KO\text{-}D10}$, n = 56. *p-value < 0.05, ** < 0.01 (two-sided Student's t-test).

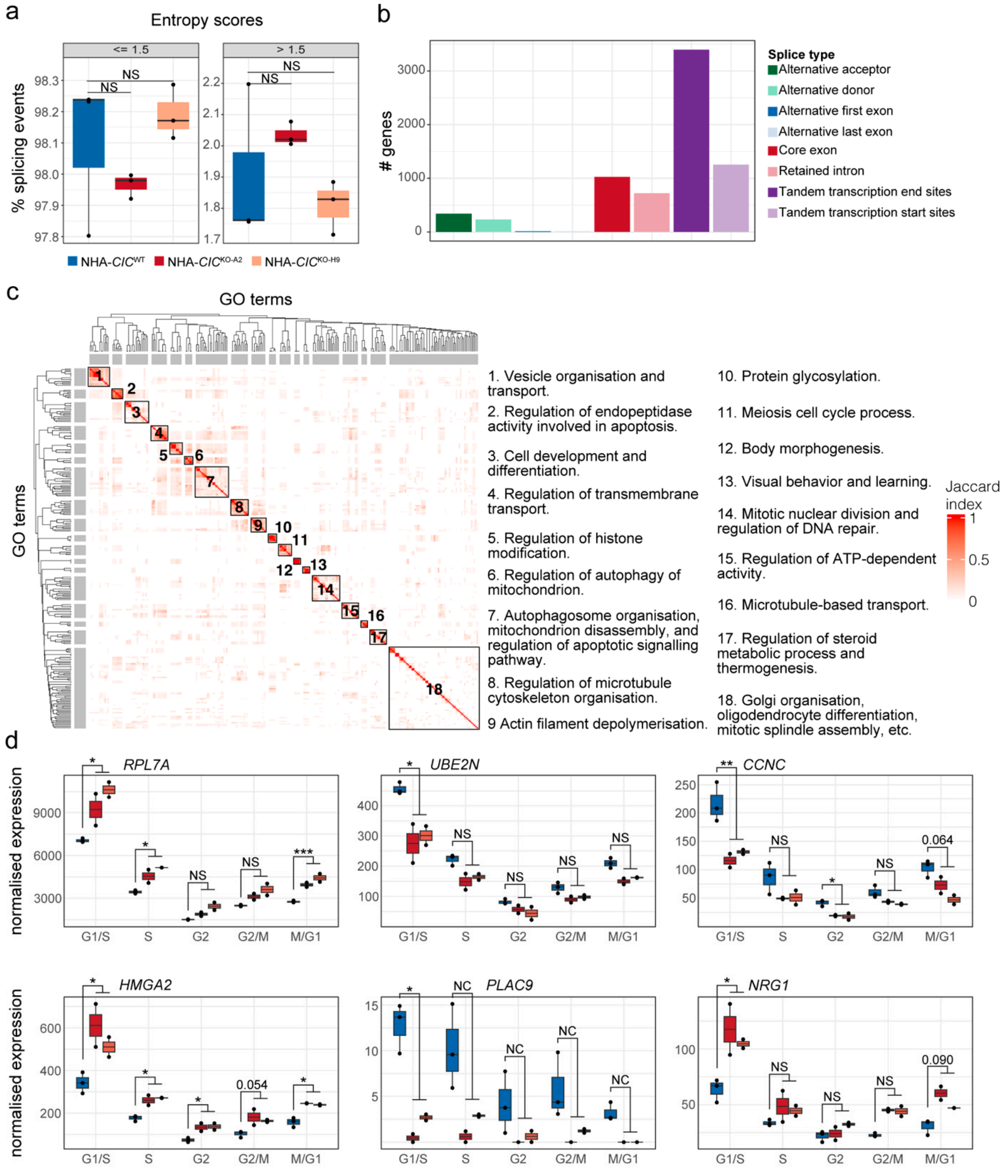

Supplementary Figure S4: Profile of alternative splicing events detected in NHA cell lines.

a. Boxplots showing the percentage of total splicing events detected with either low (<= 1.5; left panel) or high (> 1.5; right panel) entropy scores. Each point represents one of three replicates sequenced from each NHA cell line. NS indicates ANOVA followed by Tukey's tests that were not significant (p-value > 0.05). **b**. The total number of genes in which differential splicing events were detected between NHA-*CIC*^{WT} and

NHA-*CIC*^{KO} cell lines. The splicing events counted includes both significant and non-significant events categorised by type of splicing event. **c**. Heatmap showing Jaccard index similarities between 237 GO terms associated with genes containing significant differential splice events (left). The Jaccard index-based hierarchical clustering was used to summarise the GO terms into 18 groups based on similarity of gene sets associated to the terms, and these functions were summarised in the text (right). **d.** Expression of genes with differential splicing events in RNA-seq data that were also found to be differentially expressed in at least one cell cycle phase in scRNA-seq data. Normalised expression of indicated genes (scRNA-seq data) is shown across cell lines and phases. *BH-adjusted p-value < 0.1, ** < 0.01, *** < 0.001 (Wald test as implemented by DESeq2), NS: non-significant, NC: adjusted p-value not calculated due to low expression (Methods).

Supplementary Tables

Supplementary Table S1: *CIC*'s co-essential and co-expressed gene partners. a Pearson correlation coefficients were calculated between fitness scores of *CIC* and each targeted gene from DepMap KO screens. *CIC* co-essential genes were considered as those with a coefficient above the inflection point (0.178) and a BH-adjusted p-value < 0.05 (see Section 2 for more detail). **b** Co-expression z-scores derived from COXPRESdb's human Illumina-based RNA-seq data sets.

Supplementary Table S2: *CIC* mutation characteristics in DepMap 20Q1 cancer cell lines. a-b Annotations from the DepMap 20Q1 mutations dataset (CCLE_mutations.csv file; **a**) and copy number dataset (CCLE_gene_cn.csv; **b**) for all *CIC* mutations identified across cancer cell lines. Details about the annotations can be found in Ghandi et al. [92]. The "CIC isoform" column indicates whether a mutation is in a region specific to either the CIC-L or CIC-S isoforms, or a region common to both. **c** *CIC* mutant group status of all DepMap 20Q1 cancer cell lines (see Section 2 for mutant group selection criteria).

Supplementary Table S3: In silico genome-wide screening results comparing *CIC*^{HomDel} mutant cell lines to *CIC*^{WT} control cell lines. a Results obtained from lethality probability comparisons between *CIC*^{HomDel} and *CIC*^{WT} lines. P-values were obtained using Mann-Whitney U tests. Adjusted p-values were calculated using permutation tests with 10,000 random samplings. **b** Candidate *CIC* genetic interactors, defined as genes with significant differential lethality probabilities (adjusted Mann-Whitney U test p-value < 0.05 and median lethality probability > 0.5 in at least one group). **c** GO term annotations for candidate *CIC* genetic interactors shown in b (ClusterProfiler output) and cluster assignment that groups similar terms together (see Supplementary Methods).

Supplementary Table S4: Genes and proteins identified in the distinct multi-omic analyses in this study. a Summary table indicating whether a gene or its encoded protein was detected by the corresponding -omics assay indicated by the column name. *CIC* coessential gene: *CIC*'s predicted co-essential gene partners (Supplementary Table S1). *CIC* Genetic Interactor: *CIC*'s synthetic lethal genes predicted using DepMap *CIC*^{HomDel} cell lines (Supplementary Table S2). HEK IP-MS: proteins detected in CIC IP-MS data from HEK-*CIC*^{WT} cells (Supplementary Table S5). NHA IP-MS: proteins detected in CIC IP-MS data from NHA-*CIC*^{WT} cells (Supplementary Table S6). scRNAseq differentially expressed genes: Genes that were identified as differentially expressed in at least one cell cycle phase in scRNA-seq analysis comparing NHA-*CIC*^{KO} versus NHA-*CIC*^{WT} cells (Supplementary Table S7). NHA CIC ChIP-seq: high-confidence CIC ChIP peaks identified in Lee et al. [24] (Supplementary Table S9). mESC CIC ChIP-seq: CIC ChIP peaks identified in mESCs profiled by Weissmann et al. [22] (Supplementary Table S10). Alternatively spliced: genes identified as having differential splicing patterns

in NHA-CIC^{KO} versus NHA-CIC^{WT} cells (Supplementary Table S11). Total # of assays detecting gene: Tally of the number of analyses in which a gene or its encoded protein was detected.

Supplementary Table S5: Candidate nuclear interactors of CIC in HEK-*CIC*^{WT} cells. a IP-MS results for candidate CIC interactors identified in at least two of four replicate experiments performed in HEK-CIC^{WT} cells (ordered alphabetically). Mascot score: ion score for an MS/MS match based on the calculated probability (see Supplementary Methods). Total peptides: total number of peptides observed in each IP-MS experiment. Discrete peptides: number of unique peptides observed in each IP-MS experiment. IP: replicate experiment from which data are shown (E1-3: endogenous CIC replicates 1-3, M-1: ectopic MYC-tagged CIC; see Methods). **b** Enrichment analysis results for candidate interactors shown in a (ClusterProfiler output) with cluster assignment grouping similar terms together (see Supplementary Methods).

Supplementary Table S6: Candidate nuclear interactors of CIC in NHA-*CIC*^{WT} cells. a Trigger peptides synthesised for the NHA-CIC^{WT} IP-MS experiment. **b** IP-MS results for NHA-CIC^{WT} cells. Proteins detected with a $\log_2$FC > 0 were considered candidate CIC interactors. PSMs: number of total peptides observed. PEPs: number of unique peptides observed. IgG1-3: protein signal intensity of input control replicates. IP1-3: protein signal intensity of endogenous CIC IP replicates. $\log_2$FC: $\log_2$-transformed IP/IgG ratio. **c** High-confidence candidate CIC interactors that were identified in both HEK-CIC^{WT} cells (Supplementary Table S4a) and NHA-CIC^{WT} cells (b). **d** Enrichment analysis results for high-confidence candidate interactors shown in c (ClusterProfiler output) with cluster assignment grouping similar terms together (see Supplementary Methods).

Supplementary Table S7: scRNA-seq phase-specific DE results. Results (DESeq2 outputs) are shown for NHA-CIC^{KO} versus NHA-CIC^{WT} comparisons. Base mean: mean of normalised counts for all samples. SE: standard error. Stat: Wald statistic. Adjusted *p*-values were obtained using the Benjamini-Hochberg method. Genes without adjusted *p*-values were filtered by automatic independent filtering for having a low mean normalised count (see Section 2).

Supplementary Table S8: scRNA-seq phase-specific GSEA results. Results (clusterProfiler outputs) are shown for GSEAs performed on genes ranked according to their differential expression between NHA-CIC^{KO} and NHA-CIC^{WT} cells.

Supplementary Table S9: High-confidence CIC ChIP peaks identified in Lee et al. [24]. Genomic coordinates refer to the GRCh37 (hg19) assembly. Peaks were identified using MACS2. Fold-enrichment: read count fold-change between CIC ChIP and matched input. Peaks were annotated using ChIP-seeker, and the nearest gene is indicated. Peaks that overlap with peaks identified by Weissmann et al. [22] (≥ 1bp) are also indicated.

Supplementary Table S10: CIC ChIP peaks identified in mESCs. Genomic coordinates refer to the GRCh37 (hg19) assembly. Peaks were identified using MACS2 using data from Weissmann et al. [22] (see Section 2). Fold-enrichment: read count fold-change between CIC ChIP and matched IgG input. Peaks were annotated using ChIP-seeker, and the nearest gene is indicated. Overlaps with SWI/SNF peaks (identified using data from Gatchalian et al. [51]) are indicated. HGNC symbols are indicated for genes that have human orthologues, as is whether the gene was found to have a nearby CIC ChIP-seq peak in NHA cells [24].

Supplementary Table S11: Alternative splicing events detected in NHA cell lines. a Results of all splicing events quantified (Whippet.quant.jl output) in the NHA-CIC^{WT} , NHA-$CIC^{KO\text{-}A2}$, and

NHA-*CIC*$^{KO-H9}$ lines. **b** Results (Whippet.diff.jl output) of differential splicing events detected between the NHA-*CIC*^{WT} line and NHA-*CIC*^{KO} lines. Significant splicing events events were those with absolute Delta $\Psi > 0.1$ and probability > 0.9. **c** GO terms associated with genes affected by significant splicing events (b) were annotated using CluterProfiler. Those with p-values < 0.05 are shown. **d** scRNA-seq phase-specific DE results for the 51 genes affected by significant differential splicing events.

Supplementary Table S12: Phase-specific genes identified by Whitfield et al. [41] used to score and assign cells to cell cycle phases. Gene names from the original study are shown, as well as updated gene names and Ensembl gene IDs used for our data.

Supplementary Table S13: Antibodies used in this study.
